# Immunosuppressant drugs and quality-of-life outcomes in kidney transplant recipients: An international cohort study (EU-TRAIN)

**DOI:** 10.3389/fphar.2023.1040584

**Published:** 2023-04-27

**Authors:** François R. Girardin, Anna Nicolet, Oriol Bestard, Carmen Lefaucheur, Klemens Budde, Fabian Halleck, Sophie Brouard, Magali Giral, Pierre-Antoine Gourraud, Béatrice Horcholle, Jean Villard, Joachim Marti, Alexandre Loupy

**Affiliations:** ^1^ Division of Clinical Pharmacology, Department of Laboratory Medicine and Pathology, Lausanne University Hospital, Faculty of Medicine, University of Lausanne, Lausanne, Switzerland; ^2^ Division of Clinical Pharmacology and Toxicology, Department of Anesthesiology, Clinical Pharmacology, Intensive Care and Emergency Medicine, Geneva University Hospitals, Geneva, Switzerland; ^3^ Center for Primary Care and Public Health (UniSanté), University of Lausanne, Lausanne, Switzerland; ^4^ Department of Nephrology and Kidney Transplantation, Vall d’Hebron Hospital Universitari, Vall d’Hebron Institut de Recerca (VHIR), Vall d’Hebron Barcelona Hospital Campus, Universitat Autònoma de Barcelona, Barcelona, Spain; ^5^ Kidney Transplant Department, Saint Louis Hospital, Unité de Recherche Clinique, Assistance Publique-Hôpitaux de Paris, Paris, France; ^6^ Paris Translational Research Center for Organ Transplantation, Institut National de la Santé et de la Recherche Médicale UMR-S970, Université de Paris, Paris, France; ^7^ Department of Nephrology and Intensive Care, Charité-Universitätsmedizin Berlin, Corporate Member of Freie Universität Berlin and Humboldt-Universität zu Berlin, Berlin, Germany; ^8^ Department of Nephrology and Intensive Care, Charité Virchow Clinic, University Hospital, Berlin, Germany; ^9^ Nantes Université, INSERM, CRT2I—Center for Research in Transplantation and Translational Immunology, Nantes, France; ^10^ Transplantation Immunology Unit, National Reference Laboratory for Histocompatibility, Geneva University Hospitals and University of Geneva, Geneva, Switzerland; ^11^ Kidney Transplant Department, Necker Hospital, Assistance Publique-Hôpitaux de Paris, Paris, France

**Keywords:** immunosuppressant, kidney transplant patient, quality of life, PROMS, VAS (analog visual scale), EQ5D 3L, transplantation, international cohort study

## Abstract

**Introduction:** Patient-Reported Outcomes (PRO) integrate a wide range of holistic dimensions that arenot captured within clinical outcomes. Particularly, from induction treatment to maintenance therapy, patient quality-of-life (QoL) of kidney transplant recipients have been sparsely investigated in international settings.

**Methods:** In a prospective, multi-centric cohort study, including nine transplant centers in four countries, we explored the QoL during the year following transplantation using validated elicitation instruments (EQ-5D-3L index with VAS) in a population of kidney transplant patients receiving immunosuppressive therapies. Calcineurin inhibitors (tacrolimus and ciclosporin), IMPD inhibitor (mycophenolate mofetil), and mTOR inhibitors (everolimus and sirolimus) were the standard-of-care (SOC) medications, together with tapering glucocorticoid therapy. We used EQ-5D and VAS data as QoL measures alongside descriptive statistics at inclusion, per country and hospital center. We computed the proportions of patients with different immunosuppressive therapy patterns, and using bivariate and multivariate analyses, assessed the variations of EQ-5D and VAS between baseline (i.e., inclusion Month 0) and follow up visits (Month 12).

**Results:** Among 542 kidney transplant patients included and followed from November 2018 to June 2021, 491 filled at least one QoL questionnaire at least at baseline (Month 0). The majority of patients in all countries received tacrolimus and mycophenolate mofetil, ranging from 90.0% in Switzerland and Spain to 95.8% in Germany. At M12, a significant proportion of patients switched immunosuppressive drugs, with proportion varying from 20% in Germany to 40% in Spain and Switzerland. At visit M12, patients who kept SOC therapy had higher EQ-5D (by 8 percentage points, *p* < 0.05) and VAS (by 4 percentage points, *p* < 0.1) scores than switchers. VAS scores were generally lower than EQ-5D (mean 0.68 [0.5–0.8] vs. 0.85 [0.8–1]).

**Discussion:** Although overall a positive trend in QoL was observed, the formal analyses did not show any significant improvements in EQ-5D scores or VAS. Only when the effect of a therapy use was separated from the effect of switching, the VAS score was significantly worse for switchers during the follow up period, irrespective of the therapy type. If adjusted for patient characteristics and medical history (e.g., gender, BMI, eGRF, history of diabetes), VAS and EQ-5D delivered sound PRO measures for QoL assessments during the year following renal transplantation.

## 1 Introduction

Kidney transplantation remains the treatment of choice for chronic renal failure. Monitoring procedures and indicators after organ transplantation generally include surgical suite, long-term survival, and complication rates. Monitoring quality-of-life (QoL) is gaining importance as complementary outcome measures, especially because of the need of real-world data on patient wellbeing and intense resource utilization. Clinicians, researchers, and health authorities acknowledge the importance of considering patient-reported outcomes (PROs) alongside biomarkers or genetic characteristics, as multidimensional aspects of individualized treatments and for further health technology assessment (HTA) purposes. Research into health services recently focused on improving patients’ health-related QoL, particularly if long-term and expensive therapies with narrow therapeutic index are used: standardized and validated elicitation instruments are needed to derive patient-reported outcome measures (PROMs). PROMs integrate a wide range of multidimensional effects related to the initiation of immunosuppressive drugs and maintenance protocols, including health utility indexes. However, they have been sparsely considered before and after transplantation in international cohort studies, including kidney transplant recipients (KTR). Principal goals of the EU-TRAIN consortium regarding PROMs are: to provide multidimensional findings for translation to end users (clinicians and KTR), to address unmet needs on new biomarker-guided therapies, and to fill the gap related to the preponderant role of immune-suppressants on QoL.

There are disease-specific questionnaires developed for transplant patients or individuals with chronic renal failure, such as the Modified Transplant Symptom Occurrence and Symptom Distress scale derived from 59 items (MTSOSD-59R) (Kim and Jang, 2020) or the Kidney Disease and Quality-of-Life (KDQOL-36) (Chong et al., 2018). The implementation of such elicitation instruments in a routine QoL survey during follow-up (FU) visits remained difficult to achieve in larger scale, due to the number of items, language issues, and nuances between proposals in the questionnaires.

This first study aims to describe QoL in a multi-centric population of patients receiving immunosuppressive therapies to sustain kidney transplantation and contain organ rejection, by implementing PROMs based on validated short questionnaires, such as the EQ-5D-3L index and the Visual Analogue Scale (VAS) score.

## 2 Materials and methods

### 2.1 Participants

The EU-TRAIN (EUropean TRAnsplantation and Innovation) prospective cohort of kidney transplant patients is a Consortium for Research and Innovation Framework Programme H2020 that includes four countries (France, Germany, Spain, and Switzerland) and nine transplantation centers based in university hospitals.

Briefly, EU-TRAIN (https://eu-train-project.eu/) was an international, multicenter, prospective trial aiming at implementing the use of clinical decision support system to 1) evaluate non-invasive biomarkers in peripheral blood predicting anti-donor immunological activation, to 2) monitor the risk of transplant rejection without invasive procedures and measure improvement in therapy response after kidney transplantation. Eventually, we aim to assess the effectiveness and QoL and, ultimately, cost-effectiveness of the new diagnostic and monitoring approaches to improve productive and allocative efficiency in European healthcare systems.

More specifically, the primary objectives were 1) the stratification of KTR using non-invasive biomarkers for the risk of allograft rejection in the first year post transplant; 2) the re-classification of rejection diagnoses (SOC histopathology procedures) by the gene expression profiling in allograft biopsies (“Low-risk” and “High-risk” clusterings).

From November 2018 to June 2021, the total patient population included 542 KTR, out of which 491 KTR categorized by age, gender, current medications, physical characteristics (e.g., weight, height), medical history, estimated Glomerular Filtration Rate (eGFR) that determines the stage of kidney disease and the type of allograft donor. A wide range of non-invasive biomarkers will be prospectively assessed, such as T- and B-cell ELISpot assays, donor specific antibodies, blood targeted transcriptional profiling, donor-derived cell-free DNA (liquid biopsy), and ultimately AI-based predictors (e.g., algorithms, machine learning). Main indications to KT were glomerulopathy 19% (*n* = 104), polycystic kidney disease 14% (*n* = 75), chronic interstitial nephropathy 13% (*n* = 69), vascular nephropathy 12% (*n* = 63), and mixed origins 10% (*n* = 55). Further etiologies were post-renal diseases 5% (*n* = 26), diabetes 4% (*n* = 23), IgA nephropathy 4% (*n* = 23), and malformative nephropathy 4% (*n* = 20). All other causes represented 15% (*n* = 84).

The number of living donors were 107 (20%), 457 KTR (84%) had dialysis before kidney transplantation, and the average duration of dialysis was 3.3 years (min. 0.1 - max. 35 years).

During the 3 months following KT (M3), the rate of biopsies was 60% (*n* = 327) and biopsy proven acute rejection (BPAR) was 7% (*n* = 24). Between M3 and 12 months (M12), the rate of performed biopsy was 61% (*n* = 330) and BPAR was 6% (*n* = 21). CMV (Cytomegalovirus) reactivation was found in 11% of KTR (*n* = 60). The rate of BK virus (BKV) reactivation was 5% (*n* = 28) and BKV-associated nephropathy was found in 4% of KTR (*n* = 22). At M12, the total number of reported infections (outside BKV and CMV) was 821, the gastrointestinal events 361, and the total number of adverse drug events (ADE) was 3,553 (antibiotics and antifungal medications were the main agents responsible for ADE, *n* = 755).

Some indicators were not available from the KTR cohort due to the missing observations related to the COVID-19 pandemic and the relatively short observational period.

Local institutional ethics committee approvals were obtained for all nine centers.

### 2.2 Instruments

We used EQ-5D-3L instrument with permission from the EuroQol Group and VAS scale to measure patients’ QoL (Rabin and de Charro, 2001; Rabin et al., 2014). The EQ-5D-3L provides a simple description of patient self-perceived health status covering five health dimensions: Mobility, self-care, usual activities, pain/discomfort and anxiety/depression, with three response options (no problems, some problems, and severe problems). The patient response is transformed into a code with underlying value ranging from perfect health to worst possible health, and the EuroQol Group has already developed a methodology for eliciting value sets for the 3L version in most European countries. We used the value sets for France (Chevalier and de Pouvourville, 2013), Spain (Badia et al., 2001) and Germany (Greiner et al., 2005) in this study to derive EQ-5D scores. Whilst there is no EQ-5D-3L value set available for French-speaking part of Switzerland (Geneva), we used the value set from France as we considered it the most comparable to the patient and hospital settings in Geneva.

The self-reported VAS measures the patient health state and general wellbeing on a scale from 0 to 100, where 0 reflects the worst imaginable health status and 100 the best health status. It is a health summary score used in the clinical and economic evaluation of healthcare as well as in population health surveys (Dolan, 1997; Kullberg et al., 2005).

### 2.3 Study medication

In this prospective observational study, no therapeutic intervention was assessed. KTR received immunosuppressants after transplantation according to immunosuppressive protocols based on international standards. The following immunosuppressants were used as maintenance therapy to control graft rejection: Calcineurin inhibitors (tacrolimus (Tac) or ciclosporin (Cic), mutually exclusive prescription); IMPD inhibitors (mycophenolate mofetil (Mmf); and mTOR inhibitors (everolimus, sirolimus, mutually exclusive prescription).

Generally, KTR received first Tac, while fewer ones got Cic, together with Mmf as SOC. In cases of signs of nephrotoxicity, allograft rejection or certain infections, such as CMV) or BKV, or progression of neoplasms (Iaria et al., 2007), immunosuppressant therapies were switched or mTor inhibitor was added as a second line treatment.

### 2.4 Procedure

Elicitation of EQ-5D and VAS estimates were collected at baseline (M0, <24 h before transplantation) and after 1 year (M12). Validated EQ-5D in four languages (English, French, German, Spanish) were used. To ensure harmonization per protocol between countries and transplantation centers, a common eCRF (electronic case report form) was designed and developed by Consortium members. Data was entered by the principal investigators or sub-/co-investigators in the electronic case report form (eCRF), and patient data was anonymized on the electronic case report form (eCRF). Only authorized persons (principal investigators and sub-/co-investigators) were able to access the eCRF at the study sites.

### 2.5 Data analysis

We derived QoL based on data from EQ-5D and VAS, measured at inclusion (month M = 0) and at FU visit (M12), alongside descriptive statistics at inclusion, per country and hospital center. We calculated the proportions of KTR with different immunosuppressive therapy patterns and non-missing observations at baseline and at FU visit (month M = 12), taking into account those who switched to other therapies over the course of 1 year. We assessed the variation of EQ-5D and VAS scores between baseline (at inclusion) and FU visit (M12).

Finally, we investigated associations between QoL measures (EQ-5D and VAS) and types of therapies using generalized linear models, estimated in the FU visit (M12) (GLM, family binomial, link logit, Stata software, 17.0). The unadjusted model results were presented alongside results adjusted for potentially important background explanatory variables: gender, history of diabetes, body mass index (BMI), and estimated renal function at M12. The results of all models were transformed to average marginal effects for ease of interpretation. Average marginal effects show how, on average, a dependent variable (VAS or EQ-5D in our case) changes when the levels of the explanatory variables change (or at a one-unit change of the explanatory variables). Additionally, we explored whether the improvement in QoL over the course of 12 months (measured by EQ-5D or VAS) was associated with immunosuppressive therapies, taking into account cases of switching to other therapies. We used logit models (Stata software, 17.0) for both elicitation instruments (EQ-5D and VAS), with binary dependent variable taking the value of 1 if the QoL measure increased at FU visit M12 compared to baseline, and 0 otherwise. The results of the logit model (unadjusted and adjusted) were reported in odds ratios.

## 3 Results

### 3.1 Sample characteristics

Among 542 KTR included in the EU-TRAIN trial, we received individual patient data from 491 KTR who completed at least one QoL questionnaire at initiation or baseline (Month 0 = M0), whereby the French hospitals collected information on the majority of the study sample (71%, *n* = 349) (Table 1). Overall, 286 KTR completed only the VAS questionnaire (273 KTR with non-missing background characteristics), 214 KTR completed only the EQ-5D questionnaire (204 with non-missing background characteristics), and 212 KTR completed both EQ-5D and VAS questionnaires.

**TABLE 1:** Descriptive characteristics of the patient sample at baseline.

	N	Age mean IQR	Gender males	Comor-bidities (yes)	Diabetes history (yes)	Smoking history (yes)	eGFR mean IQR	BMI mean IQR	^a^VAS mean; IQR	^a^EQ-5D mean; IQR
France										
*Saint Louis, Paris*	130	55.0 [44.0; 67.0]	83 (64%)	129 (99%)	34 (26%)	39 (30%)	12.6 [5.5; 10.9]	24.8 [21.7; 27.1]	0.67 [0.55; 0.8]	0.85 [0.8; 1]
*Necker, Paris*	138	56.4 [45.0; 68.0]	90 (65%)	136 (99%)	24 (17%)	35 (25%)	9.5 [6.0; 12.0]	25.1 [21.7; 28.1]	0.63 [0.5; 0.8]	0.81 [0.75; 1]
*Hôtel Dieu, Nantes*	59	58.1 [43.0; 71.0]	34 (58%)	59 (100%)	9 (15%)	32 (54%)	9.3 [7.0; 11.0]	26.1 [22.8; 29.4]	0.68 [0.5; 0.8]	0.84 [0.8; 1]
*Bicêtre, Paris*	22	58.7 [52.0; 69.0]	12 (55%)	21 (96%)	6 (27%)	5 (23%)	8.1 [7.0; 9.0]	27.7 [25.8; 30.7]	0.71 [0.6; 0.85]	0.81 [0.85; 1]
Germany										
*Charité Virchow, Berlin*	33	53.2 [44.0; 61.0]	23 (70%)	33 (100%)	4 (12%)	11 (33%)	11.7 [8.05; 14.0]	25.2 [22.5; 26.2]	0.76 [0.7; 0.84]	0.98 [1; 1]
*Charité Mitte, Berlin*	37	55.9 [48.0; 63.0]	20 (54%)	31 (84%)	1 (3%)	12 (32%)	15.0 [15.0; 15.0]	26.0 [23.0; 28.7]	0.75 [0.7; 0.9]	0.86 [0.89; 1]
Spain										
*Bellvitge, Barcelona*	48	61.7 [53.0; 69.5]	34 (71%)	48 (100%)	18 (38%)	8 (17%)	17.0 [9.00; 25.0]	28.0 [22.7; 32.3]	0.73 [0.6; 0.8]	0.91 [0.83; 1]
*Vall d'Hebron, Barcelona*	7	61.7 [49.0; 72.0]	6 (86%)	6 (86%)	3 (43%)	4 (57%)	12.4 [9.0; 20.0]	26.6 [23.8; 27.2]	0.78 [0.7; 0.85]	0.97 [1; 1]
Switzerland										
*Geneva hospitals, Geneva*	17	55.9 [52.0; 63.0]	12 (71%)	16 (94%)	2 (12%)	7 (41%)	6.6 [5.0; 8.0]	27.1 [22.5; 30.8]	0.70 [0.6; 0.8]	0.90 [0.84; 1]
Total	491	56.7 [47.0; 68.0]	315 (64%)	479 (98%)	101 (21%)	153 (31%)	10.9 [6.2; 13.0]	25.6 [22.1; 28.7]	0.68 [0.5; 0.8]	0.85 [0.8; 1]

eGFR, estimated Glomerular Filtration Rate; BMI, body mass index; VAS, visual analog scale; EQ-5D, measure of health-related quality of life developed by the EuroQol Group with 5 Dimensions; IQR, Interquartile Range (between 25% percentile and 75% percentile).

^a^ statistical analysis of means (ANOVA) showed significant differences between countries in their scores of EQ-5D, and VAS at 1% level (F-stat. = 5.73 and F-stat. = 7.67, respectively).

The mean age ranged from 55 to 61.7 years, whereby the KTR in Spain were on average of older age than in the other countries. In all nine transplantation centers, the majority of KTR were males while the proportion varied from 54% (hospital in Germany) to 86% (hospital in Spain). The vast majority of KTR had several comorbidities (84%–100%), whilst there was a larger variation in the smoking history (17%–57%) and diabetes history (3%–43%). Mean eGFR at baseline was lowest in the University hospitals of Geneva (6.6 mL/min/1.73 m^2^) and highest in the Spanish centers (17.0 mL/min/1.73 m^2^). BMI ranged from 24.8 kg/m^2^ in France to 28.0 kg/m^2^ in Spain.

**FIGURE 1:**
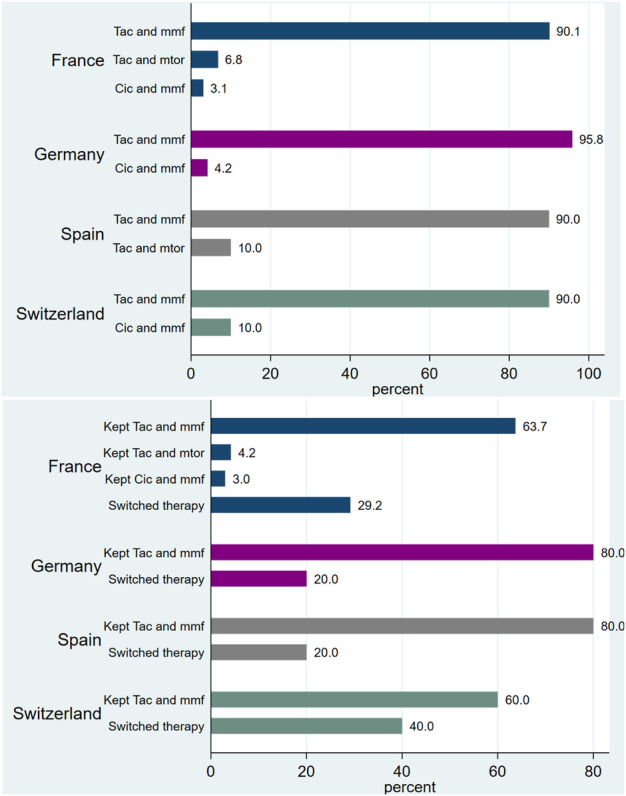
Proportion (%) of patients with immunosuppressant therapies, at baseline and at visit M12.

The proportions of KTR receiving various immunosuppression therapies at baseline and at FU visit (M12) are detailed (Figure 1). The majority of KTR in all countries (>90%) received SOC at baseline (Tac and Mmf). However, at M12, multiple KTR switched therapies, with percentages varying from 20% in Germany and Spain to 40% in Switzerland (Figure 1).

The impact of glucocorticoids on QoL was hardly assessable because they were used in high dose during the induction phase followed by tapering dosages. Therefore, their influence on patient QoL is hardly feasible without strong assumptions: 511 (94.63%) recipients had glucocorticoids after the KT with dose tapering during the study period. Thus, 415 (92%) had still low dose prednisone 5–40 mg/d after 3 months (M3) and 391 (90%) had lower dose (5–15 mg/d) after 12 months (M12).

### 3.2 Quality-of-life among KTR with various immunosuppressive therapies

**FIGURE 2:**
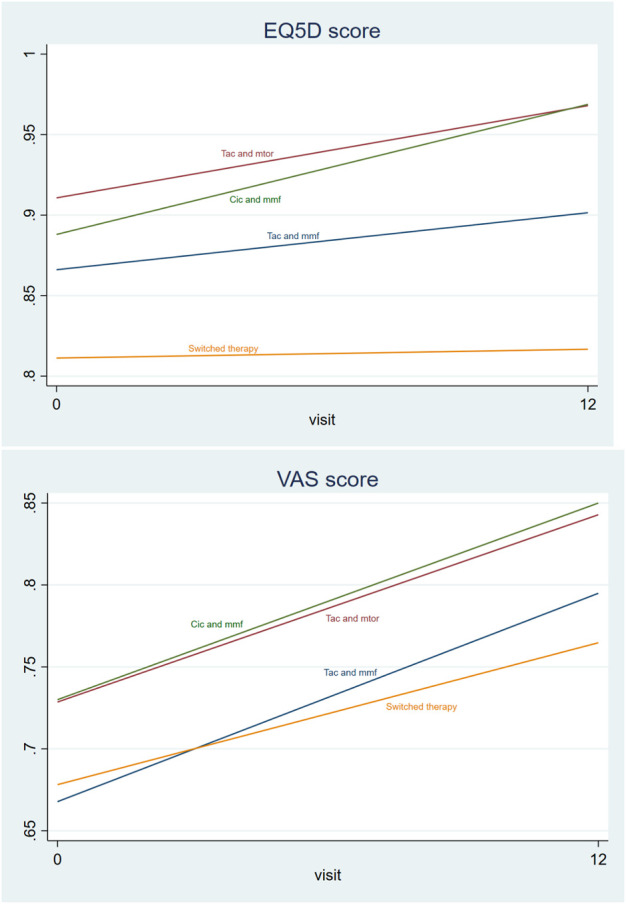
Mean EQ-5D scores and VAS scores by therapy type at baseline and in the last visit (trend) among only those people with non-missing EQ-5D and VAS scores (*N* = 205). *Overall mean EQ-5D score changed from 0.85 to 0.88, and mean VAS changed from 0.67 to 0.79

Mean VAS scores at baseline were systematically lower than EQ-5D scores, with total means of 0.68 VAS versus 0.85 EQ-5D: statistically significant differences existed between countries (Table 1). Overall, QoL measured by VAS and EQ-5D showed a positive trend over the period from baseline until the FU visit (M12) (Figure 2). KTR who switched therapies had lower EQ-5D and VAS scores than KTR keeping their therapies, especially in the case of EQ-5D (Figure 2). Additionally, VAS scores, although generally lower than EQ-5D, showed a larger increase over time for all therapy groups: mean EQ-5D score changed from 0.85 to 0.88, and mean VAS changed from 0.67 to 0.79.

Bivariate and multivariate analysis using generalized linear models showed that KTR who kept standard care therapy (Tac and Mmf) had significantly better EQ-5D and VAS scores at M12 than KTR in the group of therapy switchers, by eight percentage points (pp) in EQ-5D and four pp in VAS (Tables 2, 3). The analysis also indicated a trend for higher scores in KTR with therapies based on Tac, mTOR, Cic, and Mmf. Additionally, the eGRF was positively and significantly associated with QoL measured by EQ-5D and VAS; males tended to have higher EQ-5D score than females, and the history of diabetes was associated with a worse VAS score (Tables 2, 3).

**TABLE 2:** EQ-5D at closing visit M12 and improvement of EQ-5D over the whole observation period.

	EQ-5D, average marginal effects			Improved EQ-5D, odds ratios	
	Unadj., *N* = 214	Adj. , *N* = 204		Unadj	Adj
Type of drug			Type of drug		
***Switched therapy***	*References*		***Cic and mmf***	*References*	
*Kept Tac and mmf*	0.08**	0.06*	*Tac and mmf*	0.99	0.99
*Kept Tac and mtor*	0.13	0.06	*Tac and mtor*	1.05	1.12
*Kept Cic and mmf*	0.16	0.15	**Switched therapy**	1.21	1.27
**Males**	—	0.07**	**Males**	—	0.75
**History of diabetes**	—	−0.05	**History of diabetes**	—	1.08
**BMI**	—	0.01**	**BMI**	—	1.01
**Estimated GRF at month 12**	—	0.002***	**Estimated GRF at month 12**	—	1.01

* = *p* < 0.1, ** = *p* < 0.05, *** = *p* < 0.01.

Bold are the names of the variables used in the analysis. In bold italic is the reference category from a categorical variable *Type of drug*.

**TABLE 3:** VAS scores at closing visit M12 and improvement of VAS score over the whole observation period.

	VAS, average marginal effects			Improved VAS, odds ratios	
	Unadj., N = 286	Adj., N = 273		Unadj	Adj
Type of drug			Type of drug		
***Switched therapy***	*References*		***Cic and mmf***	*References*	
*Kept Tac and mmf*	0.04*	0.03	*Tac and mmf*	1.41	1.09
*Kept Tac and mtor*	0.07	0.04	*Tac and mtor*	1.58	1.20
*Kept Cic and mmf*	0.05	0.05	**Switched therapy**	0.43**	0.45*
**Males**	—	0.02	**Males**	—	1.13
**History of diabetes**	—	−0.07**	**History of diabetes**	—	0.94
**BMI**	—	−0.00	**BMI**	—	0.98
**Estimated GRF at month 12**	—	0.001**	**Estimated GRF at month 12**	—	1.01*

* = *p* < 0.1, ** = *p* < 0.05, *** = *p* < 0.01.

Bold are the names of the variables used in the analysis. In bold italic is the reference category from a categorical variable *Type of drug*.

Finally, although there was overall a positive trend in QoL (Figure 2, confidence intervals are presented in Appendix Table 1), the logistic regression analysis estimating the probability of improved EQ-5D or VAS during the obervational period, it did not show any significant results (Appendix Table 2). Only in specification where the effect of a therapy use was separated from the effect of switching (Tables 2, 3), the VAS score showed to be significantly worse if the KTR switched therapy during the FU period, irrespective of the immunosuppressive therapy.

**TABLE A1:** 95% Confidence intervals corresponding to the Figure 2 data points.

*Therapy/visit*	Visit 0 EQ-5D	Visit 12 EQ-5D	Visit 0 VAS	Visit 12 VAS
*Tac and Mmf*	0.87 [0.84; 0.90]	0.90 [0.87; 0.93]	0.67 [0.64; 0.70]	0.79 [0.77; 0.82]
*Tac and Mtor*	0.91 [0.82; 1.00]	0.97 [0.92; 1.02]	0.73 [0.54; 0.92]	0.84 [0.73; 0.96]
*Cic and Mmf*	0.89 [0.77; 1.00]	0.97 [0.88; 1.05]	0.73 [0.52; 0.94]	0.85 [0.73; 0.97]
*Switched*	0.81 [0.74; 0.88]	0.82 [0.74; 0.89]	0.68 [0.64; 0.72]	0.76 [0.72; 0.80]

**TABLE A2:** Improvement in raw scores (EQ-5D or VAS from baseline to visit M12, presented in odds ratios).

	EQ-5D improved		VAS improved	
	Adj. , *N* = 204	Unadj. , *N* = 214	Unadj., *N* = 286	Adj., N = 273
Type of drug				
***Switched therapy***	References		References	
*Kept Tac and mmf*	1.08	1.24	1.51	1.29
*Kept Tac and mtor*	0.92	0.99	1.52	1.26
*Kept Cic and mmf*	2.80	2.48	0.57	0.57
**Males**	0.67	-	-	0.97
**History of diabetes**	0.85	-	-	0.82
**BMI**	1.00	-	-	1.00
**Estimated GRF at month 12**	1.02**	-	-	1.01

* = *p* < 0.1, ** = *p* < 0.05, *** = *p* < 0.01.

## 4 Discussion

To our knowledge, this was the first prospective, international, multicenter study including 542 renal transplant patients that evaluated non-invasive biomarkers and immunosuppressants on PROMs. We described QoL in patients receiving immunosuppressive therapies at initiation and at (M12) and explored whether there was any improvement in QoL over the whole observation period. We found that QoL measured by VAS scores were systematically lower compared to EQ-5D and different QoL outcomes were observed at (M12) depending on the elicitation instrument (EQ-5D or VAS), and when KTR needed to switch immunosuppressants (*versus* kept standard treatment). Specifically, KTR switching therapies had lower scores in EQ-5D and VAS scores at FU visit than KTR receiving SOC (Tac and Mmf) in the first year following renal transplantation, most likely reflecting reactive changes of immunosuppressants due to adverse events. Looking at QoL improvements over the whole observation period, individuals who switched therapies were significantly less likely to improve VAS scores than non-switchers. There were no significant improvements in QoL over the observation period that was attributed to a specific treatment. Additionally, other parameters (gender, eGFR, BMI and the history of diabetes) were associated with different QoL outcomes and considered for the adjustment.

There is still a lack of common agreement regarding interpretation discrepancies between VAS ad EQ-5D values (Badia et al., 1999; Brazier et al., 2003; Lamers et al., 2006; Golicki et al., 2015). Differences in the elicitation method could provide credible explanations: the VAS provides a direct valuation of the respondent’s health state, while EQ-5D descriptive system is converted into an index score using specialized country-specific population-based value set and statistical routine (Grandy and Fox, 2008). Population-based value sets used in the current study from France, Germany, and Spain (Badia et al., 2001; Greiner et al., 2005; Chevalier and de Pouvourville, 2013) used the time trade-off (TTO) technique to elicit EQ-5D health values. TTO is a choice-based measure using hypothetical scenarios, often considered more reliable and accurate for health valuation, since it characterizes health decisions and not only health states (Dolan, 2000; Craig, 2009). Thus, differential framing and eliciting method between the VAS and TTO-based EQ-5D scores may lead to observed differences in values (Craig, 2009). Empirical studies showed evidence of a weak to moderate correlation between VAS and TTO values when performed at the same time, whilst there was a strong correlation between VAS and measures of health status (e.g., pain, physical functioning or clinical symptoms) (Bakker et al., 1994; Green et al., 2000; Lamers et al., 2006).

In this study the EQ-5D scores exceeded VAS scores, which was in line with the majority of previous studies (Brazier et al., 2003; Bernert et al., 2009; Kang et al., 2014; Burstrom et al., 2020). This finding was observed earlier as a result of disproportionate point interval, reflecting a large gap between the EQ-5D-3L values attached to poorest health state (33333) and next poorest states (e.g., 33323) (Badia et al., 1999). Such a value gap may be especially prominent in our sample of patients receiving immunosuppressive therapies after kidney transplantation who are likely to indicate poorer health states. Similarly, value gaps have been reported in other settings, such as in cardiology after acute coronary syndromes (Gencer et al., 2016; Laurencet et al., 2016) and major adverse cardiovascular events associated with COVID-19 (Tessitore et al., 2021).

We acknowledge limitations inherent to these findings issued from the EU-TRAIN cohort study. First, because of the observational nature of the study, the results did not provide any causal inference. This is particularly true if someone assumes that untoward evolution of a renal transplant might be associated with changes in immunosuppressive therapies that would fail to improve renal function or graft survival. Second, possibly for cultural reasons, the proportions of fully completed questionnaires differed significantly across centers: missing data were more frequent in Spain than in other centers. We also lacked background information about non-responders to identify any clue regarding response biases. Third, some initial therapeutic combinations are overrepresented (>90% of RTR took Tac + Mmf) and appeared to perform better according to EQ-5D: again, no inference could done be since patient selection bias could not be excluded (transplant patients will remain on initial therapy if the evolution is favorable). Fourth, in spite of mandatory therapeutic drug monitoring (TDM) requirements, the formal adherence to treatment (compliance) has not been assessed, e.g. using specific elicitation methods, such as the validated Basel assessment of adherence to immunosuppressive medications scale (BAASIS^®^) in kidney transplants (Marsicano et al., 2013). TDM data were not sufficiently detailed to assess medication compliance deviations (i.e., detailed blood sampling time with respect to drug intakes).

Strengths of the study are new insights into a wide range of medical management aspects based on PROMs, including adaptation of immunosuppressant therapy that could not be driven by laboratory parameters. Despite the initial SOC were comparable, patient characteristics and evolving trends differed across countries more than between centers. In addition, the statistical model was adjusted taking into account relevant parameters, such as the medical history and residual renal function that impacted significantly on the QoL and related health utility indexes. Finally, in line with previous studies on PROMs, we could provide evidence that VAS and EQ-5D are complementary instruments that delivered sound estimates for multidimensional FU and QoL: both elicitation methods discriminated various therapeutic outcomes, if adjusted for medical history and patient characteristics.

Future perspectives include the investigation on whether actionable data analytics could promote efficient IKR monitoring with less invasive procedures: targeted allograft protocol biopsies to predict allograft rejection based on a series of non-invasive biomarkers and other predictors are expected to facilitate patient FU, increase QoL, and reduce procedural costs.

Undoubtedly, the involvement of PROMs becomes an integral part of international cohort studies to issue recommendations in addition to clinical outcomes. Furthermore, health technology assessment (HTA) could be carried out as ancillary analisis through the development of decision models (Markov modelling, Monte-Carlo simulations, and probabilistic sensitivity analyses) to extrapolate expected effects over longer time-horizons than trials.

Beyond clinical and health economic aspects, this preliminary study lays the groundwork for future analytical frameworks to streamline pivot decision and innovation in transplantation medicine and nephrology. We expect that, on the long-term, findings derived from PROMs will help clinicians, public health authorities, and policymakers to take informed decision when revising guidance in renal transplantation standards and immunosuppression protocols.

## Data Availability

The original contributions presented in the study are included in the article/supplementary material, further inquiries can be directed to the corresponding authors.
